# Transformation of primary sensory cortical representations from layer 4 to layer 2

**DOI:** 10.1038/s41467-022-33249-1

**Published:** 2022-09-19

**Authors:** Bettina Voelcker, Ravi Pancholi, Simon Peron

**Affiliations:** 1 Center for Neural Science, New York University, 4 Washington Place Rm. 621, New York, NY 10003 USA; 2 Neuroscience Institute, NYU Medical Center, 435 E 30th St., New York, NY 10016 USA

**Keywords:** Sensory processing, Barrel cortex

## Abstract

Sensory input arrives from thalamus in cortical layer (L) 4, which outputs predominantly to superficial layers. L4 to L2 thus constitutes one of the earliest cortical feedforward networks. Despite extensive study, the transformation performed by this network remains poorly understood. We use two-photon calcium imaging to record neural activity in L2-4 of primary vibrissal somatosensory cortex (vS1) as mice perform an object localization task with two whiskers. Touch responses sparsen and become more reliable from L4 to L2, with nearly half of the superficial touch response confined to ~1 % of excitatory neurons. These highly responsive neurons have broad receptive fields and can more accurately decode stimulus features. They participate disproportionately in ensembles, small subnetworks with elevated pairwise correlations. Thus, from L4 to L2, cortex transitions from distributed probabilistic coding to sparse and robust ensemble-based coding, resulting in more efficient and accurate representations.

## Abstract (web summary)

The transform performed by superficial primary sensory cortex is poorly understood. Here the authors show that sensory stimulus-evoked activity shifts from a broadly distributed, probabilistic response in cortical layer 4 to a sparse and robust ensemble-based response in cortical layer 2.

## Introduction

Primary sensory cortices are organized into columns processing similar stimulus features and exhibiting precise interlaminar wiring^1,2^. Canonically, thalamic input mostly targets layer (L) 4, with weaker projections targeting L3 and L5^3–6^. The strongest interlaminar projection from L4 is to L3, and from L3, to L2^7,8^, making primary sensory L4 to L2 one of the earliest cortical feedforward networks. Despite extensive study, the transformation performed by this feedforward network remains unclear^9–11^.

Reduction in the proportion of neurons responding to a stimulus, or sparsification, has been proposed as a core function of feedforward processing. By concentrating the sensory response among a smaller but more reliable and robust population, sparsification is thought to facilitate perceptual readout^12–14^. The emergence of sparse but highly reliable responses has been observed across successive sensory cortical areas: for instance, in the visual ventral stream, neural responses sparsen and become increasingly object-specific^15,16^. Sparsification from L4 to L2 has been observed in many primary sensory cortices^17–19^. Despite its ubiquity, the role of this sparsification and its impact on stimulus decoding^12–14^ remains unclear.

In addition to sparse representations and expanded receptive fields, L2/3 contains groups of correlated neurons tuned to similar features, or ensembles^20–23^. In mouse L2/3, neurons with correlated activity are more likely to be directly connected^23–27^, enabling pattern completion^28,29^ and amplification^30^. Synchronous L2/3 activity in vS1 triggers strong feedback inhibition^31^, so ensembles of correlated neurons should contribute to sparseness by suppressing non-ensemble neurons in this manner^32^. Furthermore, their capacity for pattern completion and amplification could yield a more reliable neural code^22,23^. Thus, a transition to ensemble-based responses could account for many of the changes that occur from L4 to L2. Due to the dense sampling needed for studying sparse ensembles^33^, however, it remains unclear if the sparse set of stimulus-responsive neurons in superficial cortex participate disproportionately in ensembles^14,20,34^, and if they can more effectively decode sensory stimuli.

Here, we examine the transformation in vS1 touch representations from L4 to L2. In mouse vS1, thalamic input from individual whiskers projects predominantly to small, ~300 μm diameter patches of cortex known as ‘barrels’^35^. To achieve dense sampling^33^, we employ volumetric calcium imaging^36^ in transgenic mice expressing GCaMP6s in excitatory neurons^37^. Using mice with two spared whiskers performing an object localization task^36,38^, we first examine how touch receptive fields changes from L4 to L2. Next, we examine robustness and sparseness of touch responses across layers, along with the ability of neurons to decode touch features. Finally, we examine the role played by groups of correlated neurons, or ensembles, in responding to touch. Our results reveal that sensory responses become sparser from L4 to L2, primarily because responses become concentrated among a small group of highly reliable, broadly tuned neurons that participate in ensembles. These neurons are highly effective at decoding touch features. Thus, from L4 to L2, cortex transitions from a probabilistic code to an ensemble-based code with improved stimulus decoding.

## Results

### Mapping dual-whisker barrel cortex responses across layers 2–4

**Fig. 1:**
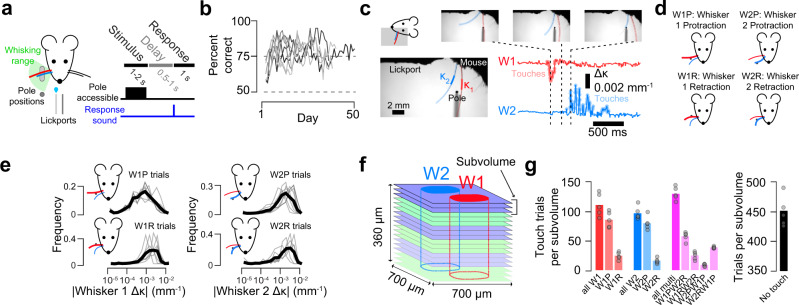
Volumetric calcium imaging during a two-whisker task. **a** Mice use two whiskers to detect a pole that appears in a proximal position range (light gray) or distal position (dark gray; Methods). Right, task timing. **b** Training progression (*n* = 7 mice). **c** Example touch video. Bottom left, the region where curvature is measured is indicated for both whiskers. Bottom right, change in curvature (Δκ) for each of the two whiskers. Moments of touch are highlighted. **d** Single-whisker touch types. **e** Distribution of curvature changes for each single-whisker touch type. Dark line, mean across mice (*n* = 7). **f** Volumetric imaging. Identically colored planes were imaged simultaneously (‘subvolume’ of 3 planes, 20 μm apart; 5-7 subvolumes per mouse). Typical barrel bounds illustrated. **g** Mean number of trials per subvolume (and, hence, neuron) for each touch type for an example animal (*n* = 5 subvolumes).

To study how neural representations change from L4 to L2, we trained transgenic mice expressing GCaMP6s in cortical excitatory neurons (Ai162 X Slc17a7-Cre)^37^ and implanted with a cranial window over vS1 on a two-whisker object localization task^30,38^ (Fig. 1a; Supplementary Table 1). On each trial, mice were presented with a pole in the whisking plane at either an accessible or out-of-reach position for a sample period (1-2 s) during which mice could palpate the pole or whisk freely. The pole was removed and, following a 0.5–1 s delay, mice were allowed to respond, receiving water for licking the right of two lickports on trials where the pole was accessible, and the left if the pole appeared out of reach. Trials typically lasted ~10 s (Methods). Mice reached stable performance (Methods; Fig. 1b) in 4.0 ± 2.1 days (mean ± S.D.; *n* = 7 mice). Changes in whisker curvature (*Δ*κ) were used as a proxy for the force impinging on the follicle base and, hence, sensory input^39^ (Fig. 1c). Our task produced four basic touch types (Fig. 1d): whisker 1 protractions (W1P), whisker 1 retractions (W1R), whisker 2 protractions (W2P), and whisker 2 retractions (W2R). The touch kinematics varied by contact type in a consistent manner across animals (Fig. 1e). Mice typically made multiple touches per trial, sometimes with both whiskers (Fig. 1c). Right licks – which are rewarded on trials where the pole is accessible and hence when touches occur – were more frequent on trials where both whiskers touched (Supplementary Fig. 1a). This was due to the higher contact forces typical of multi-whisker touch trials; right lick frequency was comparable on multi-whisker and single whisker touch trials with matched contact forces (Supplementary Fig. 1b–e). For single-whisker touches by a specific whisker, both strong retraction and protraction touches elicited high levels of rightward licking (Supplementary Fig. 1b).

Activity in well-trained mice was recorded for 18.9 ± 4.0 sessions (mean ± S.D.; range: 15 to 23, *n* = 7 mice) using volumetric two-photon calcium imaging (Fig. 1f) sampling 5–7 ‘subvolumes’ of three simultaneously imaged planes (*n* = 38 subvolumes across 7 mice). Subvolumes were imaged for 50–70 trials before proceeding to the next subvolume, with most subvolumes, and hence neurons, visited for a subset of each session (Methods). Imaging planes were aligned to a per-animal reference stack from which cortical depth and laminar bounds were determined (Supplementary Fig. 2). Barrel boundaries were obtained from the neuropil signal^36^ and visible septa in layer (L) 4 (Supplementary Fig. 3). In L2, L3, and L4, we imaged 3796 ± 1076, 5355 ± 1264 and 5372 ± 772 neurons per mouse (*n* = 7 mice), respectively (Supplementary Table 1). We obtained 820 ± 285 trials per neuron across multiple days and with a variety of touch types (Fig. 1g). Half the trials lacked touch, due to the pole being out-of-reach. Trials with touch were evenly distributed among three types: whisker 1 touch only, whisker 2 touch only, and trials where both whiskers touched.

**Fig. 2:**
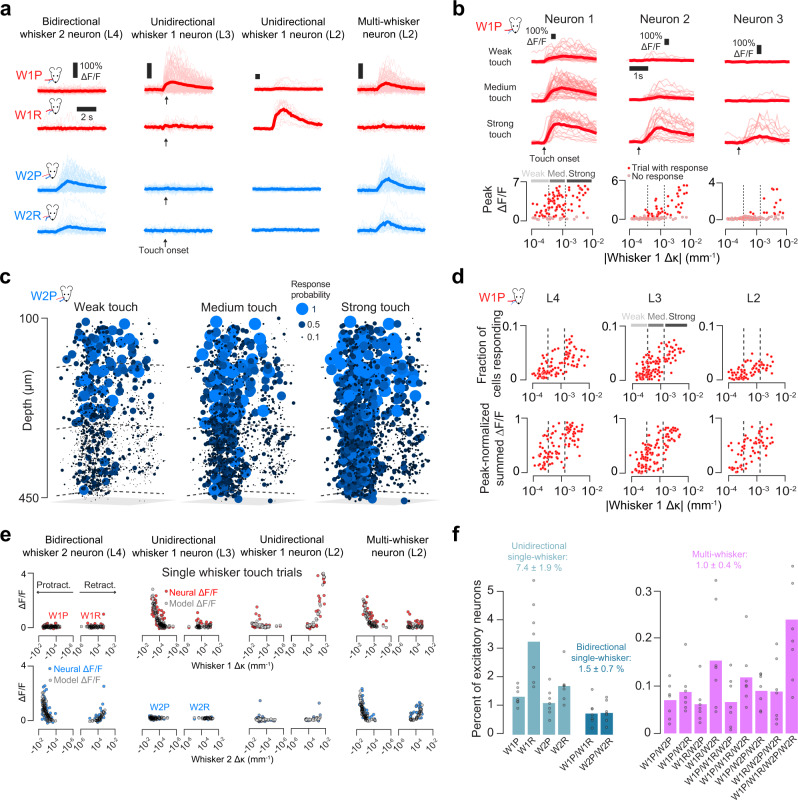
Classification of touch-sensitive neurons. **a** Example *Δ*F/F responses to all single-whisker touch types for four neurons. Thick line, mean; thin lines, individual touch responses. **b** Example responses to W1P touch for three neurons. Top, *Δ*F/F, grouped by touch intensity (weak: bottom third trial *Δ*κ; medium: middle third; strong: top third; thick lines, mean; thin lines, individual touches). Bottom, response as a function of *Δ*κ. Dark red dots, trials with detected response; gray dots, no response. Dotted vertical lines delimit the touch strength bins. **c** Response probability plotted for all neurons in an example mouse responding to weak, medium, and strong W2P touch. **d** Example population response to W1P touch. Top, fraction of neurons responding as a function of trial ∆κ (Methods). Bottom, normalized summed ∆F/F from the entire responsive pool. Dots, individual trials. Dashed vertical lines: bins for weak, medium, and strong touch trials. **e** Receptive fields for neurons in **a**. The mean *Δ*F/F as a function of trial *Δ*κ (Methods) is shown for each trial. Colored circles, real responses. Gray circles, model-predicted *Δ*F/F. **f** Frequency of different touch neuron types (*n* = 7 mice). Frequency is given for each basic touch type combination.

Individual neurons exhibited diverse touch responses, with different neurons tuned to different single-whisker touch combinations (Fig. 2a). Individual neurons showed increasing responsiveness to stronger touches (Methods; Fig. 2b, c). The fraction of neurons responding to touch also increased with touch strength, as did the aggregate response of the population (Fig. 2d). To quantify the sensitivity of neurons to touch, we used an encoding model that predicted neural activity from whisker curvature (Fig. 2e; Supplementary Fig. 4; Methods). An encoding model score was computed by measuring the Pearson correlation between the predicted and actual *Δ*F/F. Neurons were considered responsive to a given single-whisker touch type if the encoding model score exceeded both 0.1 and an activity-matched temporally shuffled response score for those trials.

Touch neurons were classified based on the combination of single-whisker touch types they responded to. Across all neurons, 9.9 ± 2.9% responded to some form of touch. We classified single-whisker touch neurons as unidirectional and bidirectional; neurons that responded on at least one type of single-whisker trial for both whiskers were classified as multi-whisker (Methods; Fig. 2a). Across all imaged neurons, 7.4 ± 1.9% of neurons responded to only one touch direction for one whisker (Fig. 2f, ‘unidirectional single-whisker’), 1.5 ± 0.7% responded to both directions for a single whisker (‘bidirectional single-whisker’), and 1.0 ± 0.4% of neurons responded to both whiskers (‘multi-whisker’).

Multi-whisker touch trials exhibited a range of inter-touch intervals (Supplementary Fig. 5). Because the initial model fit only employs touch trials where a single whisker touches the pole (Supplementary Fig. 4e), our fitting approach would miss neurons that responded exclusively to multi-whisker touch. We therefore manually examined neurons showing elevated response probability (Methods) on multi-whisker touches that were not classified as touch neurons. Only two neurons showing exclusive multi-whisker responses were found (Supplementary Fig. 5d). Thus, multi-whisker touch engages neurons that also respond to single-whisker touch.

### Touch receptive fields broaden from L4 to L2

**Fig. 3:**
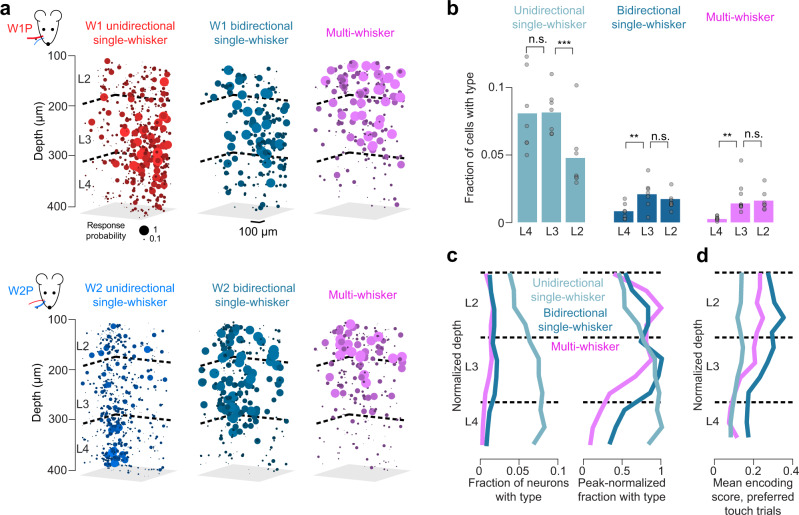
Laminar distribution of different touch cell types. **a** Example map from one mouse showing the probability of response for W1P (top) and W2P (bottom) touch. Left to right: unidirectional single-whisker neurons, bidirectional single-whisker neurons, and multi-whisker neurons. **b** Frequency of each of the major touch neuron types for L4, L3, and L2. Bars, mean (*n* = 7 mice). *P*-values indicated for paired *t*-test, ^*^p < 0.05, ^**^p < 0.01, ^***^p < 0.001; n.s. not significant. **c** Distribution of neuron types as a function of normalized depth (Methods). Left, fraction of neurons at a given depth. Right, within-type normalized fraction. **d** Encoding score as a function of normalized depth. Encoding score was calculated only across trials of the preferred type (i.e., trials with touches to which the neuron was significantly responsive; Methods).

Superficial receptive field broadening has been observed in several primary sensory cortices^40–42^; our dense sampling approach allowed us to examine this broadening at a more granular level. Unidirectional, bidirectional, and multi-whisker neurons showed distinct spatial distributions (Fig. 3a). Bidirectional neurons increased in frequency from L4 (0.008 ± 0.006) to L3 (0.021 ± 0.010; L4 vs. L3, *p* = 0.006), remaining unchanged in L2 (0.017 ± 0.007; Fig. 3b; L3 vs. L2, *p* = 0.128), as did the relative fraction of multi-whisker neurons (L4: 0.002 ± 0.001, L3: 0.014 ± 0.006, L2: 0.016 ± 0.007; L4 vs. L3, *p* = 0.001; L3 vs. L2, *p* = 0.381). The fraction of single-whisker unidirectional neurons remained unchanged from L4 (0.081 ± 0.029, mean ± S.D., *n* = 7 mice) to L3 (0.081 ± 0.016; L4 vs. L3, *p* = 0.957, paired *t*-test, *n* = 7 mice) but declined in L2 (0.048 ± 0.022; Fig. 3b; L3 vs. L2, *p* < 0.001). Thus, narrowly tuned neurons outnumber broadly tuned neurons in all layers, and broadly tuned neurons become more numerous superficially whereas narrowly tuned neurons become less numerous.

Next, we examined the distribution of touch neuron types in layer-normalized depth (Fig. 3c) finding that unidirectional neuron frequency peaks in L4, bidirectional in L3, and multi-whisker in L2. Finally, we examined changes in encoding score (Fig. 3d). Unidirectional neurons showed consistently low encoding scores. In contrast, bidirectional neurons and multi-whisker neurons showed large encoding score increases superficially, implying that responses became more reliably predictable from whisker curvature.

Noise increases with depth in two-photon microscopy^43^. To assess whether the observed receptive field broadening was impacted by the higher noise in L4, we added Gaussian noise to L2 and L3 *Δ*F/F responses to match L4 noise (Methods; Supplementary Fig. 6a–c). Following this, fewer superficial neurons were classified as touch responsive, but the trends in touch type frequencies across layers were preserved (Supplementary Fig. 6d).

Did whisker trimming and behavioral training shape the observed distribution of touch cell types? To address this, we performed lower density sampling using only 2 subvolumes per animal in a separate cohort of 5 mice over 2-3 days immediately after initial trimming as they performed a simple, touch-independent version of our task (Supplementary Fig. 7a, b; Methods). Though these ‘naïve’ mice touched less frequently, kinematics were comparable to the main dataset (Supplementary Fig. 7c, d). In naïve mice, 9.9 ± 3.0% of neurons responded to touch, comparable to mice in the main dataset (*p* = 0.368, *t*-test *n* = 7 trained mice vs. *n* = 5 naïve mice). Laminar trends in functional type were consistent with those observed in the main dataset, as were the relative frequencies of specific touch cell types (Supplementary Fig. 7e). Thus, the distribution of touch types was mostly insensitive to both trimming and training.

We next compared the distribution of unidirectional single-whisker neurons tuned to different touch directions – protraction and retraction - across layers. The fraction of retraction-preferring unidirectional neurons exceeded protraction preferring neurons in all layers, though the difference was not significant in L2 (Supplementary Fig. 8a). The decline from L4 to L2 for both protraction and retraction preferring neurons followed a similar pattern though encoding scores were slightly higher for protraction preferring neurons (Supplementary Fig. 8b, c).

### Superficial population response is sparser but more reliable

**Fig. 4:**
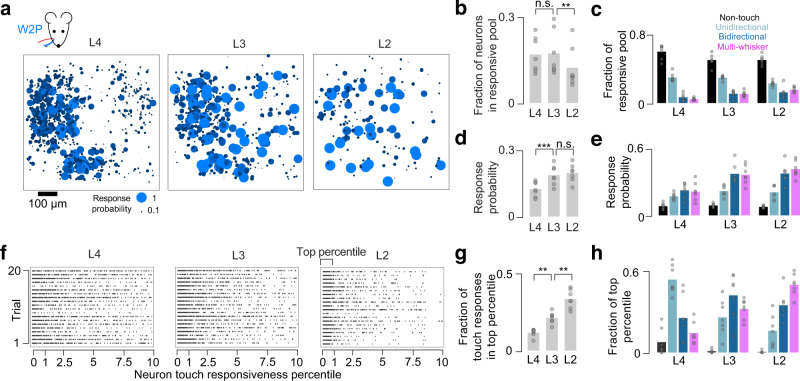
Transition to sparser, more reliable responses from L4 to L2. **a** Population response to strong W2P touches across layers. Ball size and color indicate response probability (Methods). All neurons for a layer are projected onto a single plane. **b** Fraction of neurons responding to strong W1P or W2P touches across layers (‘responsive pool’; Methods; mean, *n* = 7 mice). *P*-values indicated for paired *t*-test, ^*^p < 0.05, ^**^p < 0.01, ^***^p < 0.001, n.s not significant. **c** Fractional composition of the responsive pool in each layer by touch neuron type. **d** Response probability across layers for neurons in this responsive pool (mean, *n* = 7 mice). **e** Response probability in each layer by touch neuron type. **f** Example W1P responses for 20 strong touch trials, one trial per row, one group of 20 trials per layer. Neurons are sorted left-to-right by responsiveness percentile. A dot indicates that a particular neuron responded on a given trial. **g** Fraction of touch-evoked calcium activity that originates from the top percentile of touch-responsive neurons (mean, *n* = 7 mice). **h** Neuron types comprising the most responsive percentile of neurons.

The increase in encoding score for multi-whisker and bidirectional neurons from L4 to L2 (Fig. 3d) suggests that superficial neurons respond more reliably to touch. We, therefore, examined touch response probability across layers. Focusing on the two most numerous strong (top third of ∆κ) single-whisker touch types, W1P and W2P (Fig. 1g), we compared both the size of the responsive pool (neurons with a touch response probability exceeding the 99^th^ percentile of shuffled response probabilities for that neuron; Methods) and the response probability of neurons in this pool across layers. L4 responses to single-whisker touches were mostly confined to the barrel of the touching whisker, with many neurons exhibiting low response probability (Fig. 4a). L2 contained fewer responsive neurons, but they exhibited higher response probability and were spatially dispersed. L3 exhibited an intermediate pattern. The fraction of neurons in the responsive pool was 0.16 ± 0.05 in L4 (mean ± S.D., *n* = 7 mice) and 0.14 ± 0.05 in L3 (L4 vs. L3, *p* = 0.725, paired *t*-test, *n* = 7 mice), dropping to 0.09 ± 0.05 in L2 (Fig. 4b; L3 vs. L2, *p* = 0.003), a trend that survived L4 noise matching (Supplementary Fig. 6e). As the responsive pool shrunk, its composition changed. Though non-touch and unidirectional single-whisker (uSW) touch neurons made up a larger fraction of the responsive pool than bidirectional single-whisker (bSW) and multi-whisker (MW) neurons in all layers, the relative fraction of broadly tuned touch neurons increased superficially (Fig. 4c).

Along with this shift in composition, the probability of response among responsive pool members increased from L4 (0.13 ± 0.03; Fig. 4d) to L3 (0.21 ± 0.04; L4 vs. L3, *p* < 0.001; L2: 0.22 ± 0.03; L3 vs. L2, *p* = 0.308), a trend that remained following L4 noise matching (Supplementary Fig. 6f). The relative response probability among touch cell types also changed. In L4, response probability for touch neurons exceeded that for non-touch neurons (Fig. 4e; non-touch vs. uSW, *p* = 0.017, post-hoc Tukey’s HSD comparing neuron type pairs within a layer, *n* = 7 mice; non-touch vs. bSW, *p* < 0.001; non-touch vs. MW, *p* < 0.001), whereas responses among different touch neuron classes were similar (uSW vs. bSW, *p* = 0.177; uSW vs. MW, *p* = 0.314; bSw vs. MW, *p* = 0.984). In contrast, in L3, broadly tuned neurons became more responsive than both non-touch neurons and unidirectional single-whisker neurons (non-touch vs. bSW, p < 0.001; non-touch vs. MW, *p* < 0.001; uSW vs. bSW, *p* = 0.001; uSW vs. MW, *p* = 0.002). This relationship was preserved in L2 (non-touch vs. bSW, *p* < 0.001; non-touch vs. MW, *p* < 0.001; uSW vs. bSW, *p* < 0.001; uSW vs. MW, *p* < 0.001). Thus, superficial broadly tuned neurons are disproportionately responsive to touch.

To determine if the touch response was becoming more concentrated in the most responsive neurons, we examined the fraction of touch-evoked calcium events in the most touch responsive percentile of neurons (Fig. 4f; Methods). These neurons produce an increasing fraction of touch evoked calcium events from L4 to L3, and L3 to L2 (Fig. 4g; L4, fraction of response in top percentile: 0.13 ± 0.03, L3: 0.21 ± 0.05, L2: 0.33 ± 0.06; L4 vs. L3, *p* = 0.009, paired *t*-test, *n* = 7 mice; L3 vs. L2, *p* = 0.001). This concentration of neural response is accompanied by a shift in composition among the top percentile, with a decline in the fraction of unidirectional single-whisker neurons and an increase in the fraction of multi-whisker neurons (Fig. 4h).

Thus, the transition to a sparser representation from L4 to L2 is accompanied by the emergence of a small group of broadly tuned neurons that respond more consistently to touch.

### Decoding for higher-order stimulus features improves from L4 to L2

**Fig. 5:**
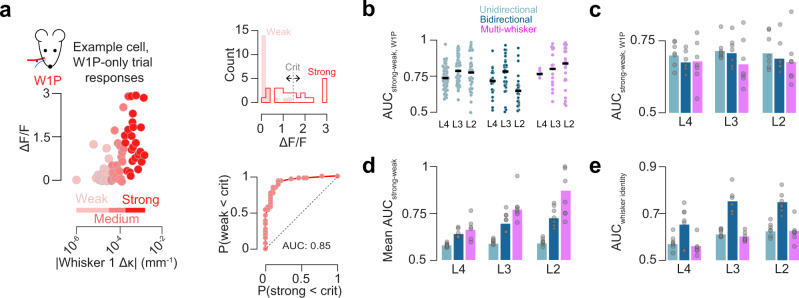
Decoding of touch features by neuron type and layer. **a** Example ROC analysis for single neuron touch force decoding (Methods). Left, per-trial peak ΔF/F as a function of *Δ*κ. Shades of red indicate force bins each containing a third of trials. Top right, histogram of strong and weak ΔF/F responses, with example criterion point for generating ROC curve. Bottom right, example neuron ROC curve. **b** Decoding for all neurons from an example mouse of a given type and layer comparing neural response to strong and weak W1P touches. Dark line, median across neurons. **c** AUC across all mice for W1P touch force. Bar indicates cross-animal mean (*n* = 7 mice). **d** As in **c**, but pooled across all four single whisker trial types. **e** Discriminability of touching whisker identity. (mean, *n* = 7 mice). ROC, receiver operating characteristic; AUC, area under the curve.

Is this superficial increase in response reliability accompanied by improved stimulus decoding? To address this, we first examined the decoding of touch force. Specifically, for a given single whisker touch type, we asked how well neural activity can distinguish the strongest touches from the weakest touches using receiver operating characteristic (ROC) analysis (Methods; Fig. 5a). For a given single-whisker touch type, multi-whisker, bidirectional single-whisker, and unidirectional single-whisker neurons performed comparably in all layers (Fig. 5b, c; L4, *p* = 0.737, ANOVA comparing three neuron types within a layer, *n* = 7 mice; L3, *p* = 0.317; L2, *p* = 0.708). Decoding of force for a single whisker did not change from L4 to L3 or L3 to L2 across any touch neuron type (Fig. 5c). In contrast, multi-whisker and bidirectional single-whisker neurons outperformed unidirectional single-whisker touch neurons in most layers when decoding ability was averaged across all four single-whisker touch types (Fig. 5d; L4: MW vs. uSW, *p* < 0.001, post-hoc Tukey’s HSD comparing neuron types within a layer, *n* = 7 mice; bSW vs. uSW, *p* = 0.010; L3: MW vs. uSW, *p* < 0.001; bSW vs. uSW, *p* = 0.028; L2: MW vs. uSW, *p* < 0.001; bSW vs. uSW, *p* = 0.113). Further, decoding of strong vs. weak touches improved substantially from L4 to L3 for bidirectional and multi-whisker neurons (bSW L4 vs. L3, *p* = 0.004; L3 vs. L2, *p* = 0.095; MW L4 vs. L3, *p* = 0.009; L3 vs. L2, *p* = 0.099).

We next asked how well individual neurons discriminate between the two touching whiskers. Here, bidirectional neurons outperformed unidirectional and multi-whisker neurons across most layers (Fig. 5e; L4: bSW vs. MW *p* = 0.027, post-hoc Tukey’s HSD, *n* = 7 mice; bSW vs. uSW, *p* = 0.066; L3: bSW vs. MW *p* < 0.001; bSW vs. uSW, *p* < 0.001; L2: bSW vs. MW, *p* < 0.001; bSW vs. uSW, *p* < 0.001). Among bidirectional single-whisker neurons, decoding improved from L4 to L3 (*p* = 0.010, paired *t*-test, bSW, *n* = 7 mice) but not L3 to L2 (*p* = 0.818).

In sum, touch neurons of any type and layer perform comparably when decoding the force for a specific single whisker touch; multi-whisker neurons are best at decoding force in a whisker-invariant manner; bidirectional single-whisker neurons are best at decoding the identity of the touching whisker. For these stimulus features, decoding improves from L4 to L2.

### Superficial broadly tuned neurons exhibit elevated functional coupling

**Fig. 6:**
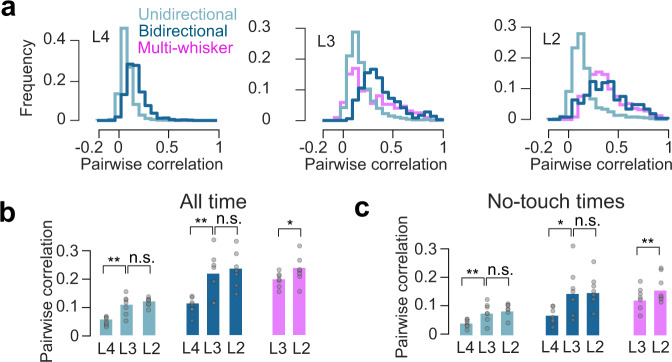
Pairwise correlations across layers and neuron types. **a** Distribution of pairwise correlations for different touch types for an example animal, computed over all imaging time. **b** Average pairwise correlations for specific neuronal types, layers. Correlations were computed using all available imaging time. Bars, mean (*n* = 7 mice). *P*-values indicated for paired *t*-test, ^*^p < 0.05, ^**^p < 0.01, ^***^p < 0.001, n.s not significant. **c** As in **b**, but epochs around touch (from 1 s prior to 10 s after) were excluded.

Touch responses sparsen superficially and become more reliable (Fig. 4), suggesting that whereas L4 neurons act independently, L3 and especially L2 neurons respond consistently as a group. To assess this, we computed pairwise correlations for each layer and touch neuron type. In L3 and L2, many bidirectional single-whisker neuron and multi-whisker neuron pairs exhibited high correlations (Fig. 6a) Broadly tuned neurons had higher pairwise correlations than unidirectional single-whisker touch neurons in L4 (Fig. 6b; bSW vs. uSW, *p* < 0.001, paired *t*-test, *n* = 7 mice), L3 (bSW vs. uSW, *p* = 0.003, Tukey’s HSD, *n* = 7 mice; MW vs. uSW, *p* = 0.016), and L2 (bSW vs. uSW, *p* = 0.001; MW vs. uSW, *p* = 0.001). In L3 and L2, bidirectional single-whisker neurons and multi-whisker neurons had comparable pairwise correlations (L3: MW vs. bSW, *p* = 0.753; L2: MW vs. bSW, *p* = 0.918). Correlations among bidirectional single-whisker neurons increased from L4 to L3 (L4 vs. L3, *p* = 0.003, paired *t*-test, *n* = 7 mice), as did correlations among unidirectional single-whisker neurons (L4 vs. L3, *p* = 0.002), but neither changed from L3 to L2 (uSW, L3 vs. L2, *p* = 0.290; bSW, L3 vs. L2, *p* = 0.226). They increased slightly for multi-whisker neurons from L3 to L2 (L3 vs. L2, *p* = 0.025).

The structure of correlations during spontaneous activity can resemble the structure of stimulus-evoked correlations, potentially due to underlying connectivity^23,44^. We, therefore, examined the correlation structure during periods of no touch by excluding all time points from 1 s before to 10 s after any touch. Though the correlations were lower than those observed when including touch, cross-laminar trends were preserved. As with correlations measured for all time, non-touch correlations increased from L4 to L3 (uSW, L4 vs. L3, *p* = 0.005, paired *t*-test, *n* = 7 mice; bSW, L4 vs. L3, *p* = 0.013), remained unchanged from L3 to L2 for single-whisker neurons (uSW, L3 vs. L2, *p* = 0.357; bSW, L3 vs. L2, *p* = 0.908), and increased slightly for multi-whisker neurons (L3 vs. L2, *p* = 0.010).

In sum, within-group correlations for all touch neuron types increase superficially. Broadly tuned neurons exhibit higher within-group correlations than narrowly tuned neurons, even during non-touch epochs.

### Superficial touch activity is concentrated in ensembles

**Fig. 7:**
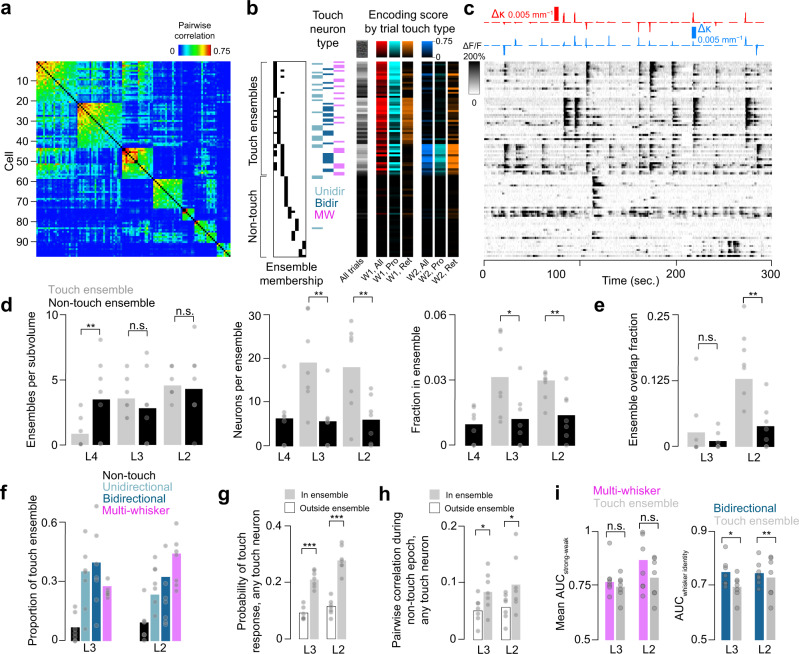
Ensembles and their role in touch responses. **a** Pairwise correlation matrix for ensemble neurons in L2 of an example mouse (Methods). Ensembles are sorted by mean within-ensemble correlation. **b** Classification of ensemble members. Left, assignment of each neuron to a particular ensemble. Middle, neural type. Right, encoding scores for given trial types. **c** ΔF/F for the neurons in **a**, **b** over the course of 5 min. Top, Δκ for whisker 1 (red) and whisker 2 (blue). **d** Basic properties of ensembles across layers. Left to right: ensembles per subvolume; number of neurons per ensemble; fraction of neurons belonging to an ensemble. Grey, touch ensembles. Black, non-touch ensembles. Bars indicate mean (*n* = 7 mice). *P*-values indicated for paired *t*-test, ^*^p < 0.05, ^**^p < 0.01, ^***^p < 0.001, n.s not significant. **e** Fractional overlap between ensembles (*n* = 7 mice). **f** Composition of touch ensembles. **g** Probability of responding on any touch for a touch neuron within (grey) or outside (white) touch ensembles (mean, *n* = 7 mice). **h** Mean pairwise correlation during non-touch epochs for touch cells within (grey) and outside (white) touch ensembles (*n* = 7 mice). **i**. Decoding by touch ensembles. Left, mean AUC (*n* = 7 mice) for decoding strong vs. weak touches. Right, mean AUC for decoding touching whisker identity.

Ensembles – groups of neurons that are co-active, often in response to a common stimulus – are frequently observed in cortex^21,23^. Given the elevated correlations among broadly tuned neurons, we asked if these neurons are more likely to participate in ensembles. We assigned neurons to ensembles based on the pairwise correlation matrix across all simultaneously imaged pairs, grouping neurons whose mutual pairwise correlations were above a threshold (Methods; Fig. 7a). Some ensembles consisted almost entirely of touch neurons, with specific ensembles preferring specific types of touch (Fig. 7b, c).

The number of ‘touch ensembles’ (ensembles where at least 50% of neurons were touch neurons) per subvolume increased from 0.9 ± 1.2 (mean ± S.D., *n* = 7 mice) in L4 to 3.6 ± 1.5 in L3 (L4 vs. L3, *p* = 0.004, paired *t*-test, *n* = 7 mice), and 4.6 ± 1.1 in L2 (Fig. 7d; L3 vs. L2, *p* = 0.086). The number of non-touch ensembles did not change, from L4 (3.7 ± 3.0) to L3 (3.0 ± 2.7; L4 vs. L3, *p* = 0.489), and L3 to L2 (4.6 ± 2.9; *p* = 0.130). Non-touch ensembles were more numerous in L4 (touch vs. non-touch, *p* = 0.020, paired *t*-test, *n* = 7 mice), but comparable in number in L3 (*p* = 0.596) and L2 (*p* = 1). Because 4/7 mice had no L4 touch ensembles, we excluded L4 from touch ensemble analyses. Touch ensembles in L3 contained 19.3 ± 10.0 neurons; L2, 18.3 ± 10.3 neurons (L3 vs. L2, *p* = 0.844). Non-touch ensembles also remained stable in size, with 6.4 ± 6.1 neurons in L4, 5.8 ± 5.8 neurons in L3 (L4 vs. L3, *p* = 0.808), and 6.1 ± 5.3 neurons in L2 (L3 vs. L2, *p* = 0.874). Non-touch ensembles were smaller than touch ensembles in L3 (non-touch vs. touch ensemble neuron count, *p* = 0.003) and L2 (*p* = 0.003). The fraction of neurons in touch ensembles did not change from L3 (0.032 ± 0.018) to L2 (0.030 ± 0.006; L3 vs. L2, paired *t*-test, *p* = 0.800). Non-touch ensemble fraction also did not change from L4 (0.010 ± 0.008) to L3 (0.012 ± 0.013; L4 vs. L3, *p* = 0.608) or L3 to L2 (0.014 ± 0.011; L3 vs. L2, *p* = 0.686). The fraction of neurons in touch ensembles exceeded the non-touch ensemble fraction in L3 (non-touch vs. touch ensemble fraction, *p* = 0.014, *n* = 7 mice) and L2 (*p* = 0.005). Thus, touch ensembles are a feature of L3 and L2, but not L4, whereas non-touch ensembles are present in all layers.

Individual neurons can belong to multiple ensembles (Fig. 7b). We quantified ensemble overlap as the size of the intersection among two ensembles divided by the size of their union. Overlap among touch ensembles increased from 0.03 ± 0.05 in L3 to 0.13 ± 0.05 in L2 (Fig. 7e; L3 vs. L2, *p* = 0.007, paired *t*-test, *n* = 7 mice); the change in overlap among non-touch ensembles was not significant (L3, overlap: 0.01 ± 0.02; L2: 0.04 ± 0.04, L3 vs. L2, *p* = 0.133). Touch ensembles exhibited more overlap than non-touch ensembles in L2 (touch vs. non-touch, *p* = 0.001), but not L3 (*p* = 0.249). Thus, touch ensemble overlap increases from L3 to L2.

We next asked whether broadly-tuned neurons are overrepresented in touch ensembles. Though they are relatively rare even in L2 (Fig. 3b), broadly tuned neurons made up a majority of ensemble neurons in both L2 and L3 (Fig. 7f). Comparing L2 and L3, we found that the fraction of ensemble neurons that were also multi-whisker neurons increased from L3 to L2 (Fig. 7f; L3 vs. L2, *p*-value=0.032, paired *t*-test, *n* = 7 mice), while the unidirectional single-whisker neuron fraction declined (Fig. 7f; L3 vs. L2, *p*-value=0.018). Thus, broadly tuned neurons constitute the majority of ensemble members, with an increase in the proportion of multi-whisker neurons from L3 to L2 at the expense of unidirectional single-whisker neurons.

Touch ensemble neurons responded more robustly to touch than touch neurons outside ensembles (Fig. 7g). The probability of any touch ensemble neurons responding to touch exceeded that of touch neurons outside touch ensembles for L3 (in ensemble: 0.21 ± 0.03, outside: 0.09 ± 0.02, *p* < 0.001, paired *t*-test, *n* = 7 mice) and L2 (in ensemble: 0.28 ± 0.04, outside: 0.11 ± 0.03, *p* < 0.001). Furthermore, touch neurons within ensembles had higher pairwise correlations during periods of no touch in both L3 (Fig. 7h, mean Pearson correlation in ensemble: 0.09 ± 0.03, outside: 0.05 ± 0.02, *p* = 0.022) and L2 (in ensemble: 0.10 ± 0.05, outside: 0.06 ± 0.02, *p* = 0.047) compared to touch neurons outside ensembles. We next asked how well touch ensemble neurons decode touch features. We found that touch ensemble neurons decoded strong from weak touches across all touch types as well as multi-whisker neurons (Fig. 7i; L3, touch ensemble vs. multi-whisker decoding, *p* = 0.871 paired *t*-test, *n* = 7 mice; L2, *p* = 0.329). When decoding touch whisker identity, however, bidirectional single-whisker neurons slightly but significantly outperformed touch ensemble neurons (Fig. 7i; L3, touch ensemble vs. bidirectional single-whisker decoding, *p* = 0.031; L2: *p* = 0.009). Thus, because ensembles contain a disproportionate number of broadly tuned neurons, they contain neurons that can effectively decode both touch strength and whisker identity.

## Discussion

We assess trans-laminar transformations of touch representations across cortical layers 2–4 of vS1 in mice performing a two whiskers object localization task. We observe distinct depth distributions for different touch neuron types: unidirectional single-whisker neurons peak in L4, bidirectional single-whisker neurons in L3, and multi-whisker neurons in L2 (Fig. 3). This is accompanied by a transition from touch responses in L4, where individual neurons from a large population respond with low probability, to sparser but more consistent responsiveness in L3 and especially L2 (Fig. 4). There, more than a third of the touch-evoked response is confined to the top 1% of responding neurons. Decoding of force across multiple whiskers as well as decoding of whisker identity improve from L4 to L2, with specific touch neuron types best decoding each feature (Fig. 5). These broadly-tuned superficial neurons form ensembles^21,23^, or groups of highly correlated neurons (Figs. 6 and 7). Thus, the transition from L4 to L2 yields sparser yet more informative and robust responses, potentially facilitating perceptual readout^12,13,34^.

Sparse activity is a common feature of sensory L2/3, yet its origin and function remain unclear^13,23,32^. Given that synchronous excitatory activity evokes strong feedback inhibition in vS1 L2/3^31,45–47^, ensemble activation should trigger feedback inhibition^13,32^, thereby confining the response mostly to ensemble members. Touch ensemble members show both robust decoding of vibrissal features and highly reliable touch responses. This suggests that ensemble-based sparsification may be a key function of superficial cortical circuitry, yielding a small population of neurons that provides a robust stimulus response well-suited for perceptual readout^12,13^.

We find that touch neurons in L2/3 of vS1, especially those with broad tuning and participating in ensembles, exhibit high pairwise correlations even during times without direct sensory input, suggesting that these neurons may be interconnected^23–25^. Several lines of evidence support this view. First, sparse populations in vS1 expressing the activity-linked immediate early gene c-*fos* exhibit elevated connectivity^48^. Second, touch evoked responses in L2/3^49^ fall within the time window of maximal synaptic potentiation^50^, so that repeated touch should drive connectivity among these neurons. Finally, following the lesion of tens of touch neurons in vS1 L2/3, the spared touch population shows a decline in responsiveness consistent with amplification due to recurrent connectivity^30^. If vS1 L2/3 touch neurons are wired in this manner, it would augment their recruitment of feedback inhibition, thus contributing to sparsification.

Superficial receptive field broadening has been observed in many primary sensory cortices^40–42^. What is the circuit basis for such receptive field broadening in superficial vS1? Spines in L2 vS1 neurons exhibit a mix of single- and multi-whisker responses^51^, but the relative contributions of intra-laminar input, input from L4^7^, or even direct thalamic input^52^ remain unknown. Given the distance-dependence of connections^53^ and the abundance of intra-laminar connections^7^, the observed spatial broadening in connectivity from L4 to L2^54^ likely contributes to the decline in single-whisker unidirectional neurons from L4 to L2 and increasing frequency of more broadly tuned neurons. At the same time, multi-whisker input from posterior medial thalamus preferentially targeting L2^55^, and even feedback from higher order areas^56^, also likely play a role. Future experiments will be needed to tease apart the contributions of specific inputs to L2/3 receptive fields.

Our results likely underestimate the fraction of neurons that respond to touch in the imaged volume. Mice in our study are trimmed to two whiskers, but we image an area spanning the barrel fields of approximately 7 whiskers (field-of-view: 700 ×700 μm; C2 barrel radius^7^: 150 μm). Multiplying our estimates of various touch type frequencies by 7/2 implies that in our field of view, unidirectional single-whisker neurons make up 28% of L4, 28% of L3 and 17% of L2; bidirectional single-whisker neurons make up 3% of L4, 7% of L3, and 6% of L2; multi-whisker neurons make up 1% of L4, 5% of L3 and 7% of L2. Such proportional scaling for all types, however, is unlikely: in anesthetized mice, direct stimulation of the eight whiskers surrounding the preferred whisker evoke responses in a large fraction of L2 touch-responsive neurons, suggesting that most touch neurons in L2 are multi-whisker^57^.

We trimmed our mice to two whiskers prior to starting behavior, imaging for several weeks once mice were well-trained. Trimming can produce both reduced^58^ and enhanced^59^ vS1 responsiveness to the spared whisker. Operant training can enhance responsiveness among reward-predictive neurons in primary sensory cortex^60^. In our case, mice trimmed immediately prior to imaging in a simplified task had comparable numbers of touch cells to well-trained mice trimmed long before imaging, with laminar trends and relative frequency across type generally preserved (Supplementary Fig. 7). Thus, neither training nor trimming impacted overall touch responsiveness in our task, consistent with previous longitudinal experiments in mice performing a single-whisker variant of our task^36^. Despite this, we cannot rule out subtler changes in touch representations due to training or trimming.

We did not observe neurons that responded exclusively to multi-whisker contacts, despite observations of enhanced responses when whiskers are stimulated in rapid succession under other contexts^61^. Such responses are likely crucial in texture processing, where slip-stick events occur as the animal’s whisker moves against a surface^62,63^. Because our task employs active touch, the animal dictates the inter-touch interval. Thus, in contrast to studies employing direct stimulation of multiple whiskers^61^ or those using texture stimuli^62,63^, mice experienced relatively few inter-touch intervals below 50 ms, the typical range where enhancement is seen.

Layers 2 and 3 are often treated as a single layer. Though many differences between L2 and L3 exist^10^, physiological and morphological changes are often gradual^54^. Consequently, even the depth of the L2-L3 boundary within vS1 has been debated, with some proposing a thinner L2^2,8^ and others using approximately equal thicknesses^7^. Dividing L2/3 equally, we find several differences between L2 and L3. First, the fraction of unidirectional single-whisker neurons declines from L3 to L2. Second, spatial intermingling among neurons tuned to different whiskers is more pronounced in L2 than L3. Third, the fraction of neurons responding to touch declines, and the fraction of touch-evoked activity occurring in the most responsive neurons increases, so that L2 touch responses are more concentrated than L3. Thus, changes in connectivity and morphology from deep L3 to superficial L2^54^ are accompanied by specific changes in sensory responses.

We show that from L4 to L2, the touch population response transitions from a diffuse and probabilistic one consisting mostly of narrowly tuned neurons to a sparse and robust one consisting mostly of broadly tuned neurons organized into ensembles. In L2/3 of mouse V1, stimulation of a small number of ensemble neurons can drive perceptual report, suggesting that small groups of such neurons can strongly influence perception^28,29^. Though we do not explicitly test the perceptual role of touch ensemble neurons, our decoding analysis suggests that feedforward processing in superficial cortex improves decoding for certain stimulus features, thereby potentially facilitating perceptual readout^12,13^.

## Methods

### Animals and surgery

Cranial windows were assembled by gluing a 3.5 mm circular #1.5 coverslip to a 4.5 mm circular #1.5 coverslip (Norland 61 glue). Windows were implanted over vS1 in P60-P90 Ai162 (JAX 031562) X Slc17a7-Cre (JAX X 023527) mice^37^ of mixed sex, as described previously^30^. In vS1, these mice express GCaMP6s exclusively in excitatory neurons. Following surgical recovery, mice were placed on water restriction. Mice were housed on a reverse light cycle lasting 12 h. Water restricted mice were typically given 1 mL/day, with adjustments to ensure weight stayed above 80% of unrestricted baseline^64^. The location in vS1 of barrels corresponding to whiskers C1-3 was identified by measuring the *Δ*F/F at coarse resolution (4X; 2.2 × 2.2 mm field of view) on a two-photon microscope while the whiskers were individually deflected. Animals were trimmed to the two whiskers whose barrels had the least obstructive vasculature, typically C2 and C3. Subsequent trimming occurred every 2-3 days. All animal procedures were in compliance with protocols approved by New York University’s University Animal Welfare Committee.

### Behavior

Water-restricted mice were handled and head-fixed to habituate them to the behavioral apparatus. Mice were trained on an object localization task^30^ in which a metal pole (0.5 mm diameter; Drummund Scientific, PA, USA) vertically moves into the range of the mouse’s whiskers either at a distal out-of-reach position or at a range of accessible proximal positions. On any given proximal trial, the pole appears at random position drawn from a range typically spanning 5 mm along the anterior-posterior axis. In all trial types, the pole remains within the whisking plane for 1-2 s, after which it moves out of reach. Pole insertion and removal is accompanied by a 50 ms white noise sound (60–70 dB) to encourage whisker movement. 0.5 s after the pole is withdrawn, an auditory cue (3.4 kHz, 50 ms) indicates to the mouse to make a response, with the left lickport rewarded on distal trials and the right lickport rewarded on proximal trials. On all trials, the lickport is withdrawn and moves into an accessible position only during the response epoch (i.e., after the auditory cue). Incorrect responses result in a timeout (5 s) and premature withdrawal of the lickport. Altogether, correct trials typically lasted 10 s whereas incorrect trials lasted 15 s, with mice averaging ~5 trials per minute. Mice were considered to reach criterion performance once d-prime exceeded 1.5 for two consecutive days. For naïve mice (Supplementary Fig. 7a), the pole always appeared in the proximal position range and only one lickport was used, so that mice received water on nearly all trials. Thus, this simplified task did not require the mouse to attend to the pole.

### Whisker videography

Whisker video was acquired using custom MATLAB (version 2019a; MathWorks) software from a CMOS camera (Ace-Python 500, Basler) running at 400 Hz and 640 × 352 pixels and using a telecentric lens (TitanTL, Edmund Optics). Illumination was via a pulsed 940 nm LED (SL162, Advanced Illumination). 7–8 s of each trial were imaged, including 1 s prior to pole movement, the period when the pole was in reach, and several seconds after the pole was retracted. Data was processed on NYU’s High Performance Computing (HPC) cluster: first, candidate whiskers were detected using the Janelia Whisker Tracker^65^. Next, whisker identity was refined and assessed across a single session using custom MATLAB software^30,36^. Following whisker assignment, curvature (κ) and angle (θ) were calculated at specific locations along each whisker’s length. Change in curvature, *Δ*κ, was calculated relative a resting angle-dependent baseline curvature value obtained during periods when the pole was out of reach. Next, automatic touch detection was performed. Touch assignment was manually curated using a custom MATLAB user interface^30^.

With the exception of our touch-detection algorithm, analyses, including model fitting, employed a down-sampled version of *Δ*κ to match the sampling rate of the calcium imaging data (7 Hz vs. 400 Hz). Specifically, we used the maximal | *Δ*κ | value over the ~140 ms duration of a single imaging frame, while preserving the sign. To obtain a single *Δ*κ value for a trial, we computed the mean across all time points for that trial during which the whisker is touching the pole. To compare *Δ*κ across whiskers and between retractions and protractions (Supplementary Fig. 1), we normalized *Δ*κ for all touches of a given single-whisker touch type by dividing with the 95th percentile of per-trial values. Where applicable, trials were partitioned into equal sized thirds based on this value, resulting in ‘strong’, ‘medium’, and ‘weak’ touch trial groupings. (Fig. 1g).

### Two-photon imaging

Imaging was performed using a MIMMS (http://openwiki.janelia.org/wiki/display/shareddesigns/MIMMS) two-photon microscope with a 16X objective (Nikon). Illumination was at 940 nm (Chameleon Ultra 2; Coherent), with power rarely exceeding 50 mW. Three imaging planes spanning 700-by-700 μm (512-by-512 pixels) and spaced 20 μm apart (‘subvolume’) were acquired at a ~7 Hz. Depth was modulated with a piezo (P-725KHDS; Physik Instrumente). Power was depth-adjusted in software with an exponential length constant having a value of 250 μm. Imaging data was acquired using Scanimage (version 2017; Vidrio Technologies).

Each of 5-7 subvolumes was imaged for 50-70 trials, followed by the next subvolume, and so on. Most subvolumes were imaged on any given day. After the first imaging day, motion-corrected mean images were collected for each plane and used as reference images on subsequent days.

Imaging data were processed on the NYU HPC cluster immediately after acquisition, as described previously^36^. The first step was motion correction via image registration. Next, for the first day of imaging, neurons were detected using an automated algorithm based on template convolution. This initial segmentation was manually curated, and a reference segmentation was established for that plane. On subsequent imaging sessions, the reference segmentation was algorithmically transferred to the new data^66^. Following segmentation, neuropil subtraction and *Δ*F/F computation were performed. For most analyses, the *Δ*F/F trace was used.

### Layer assignment

For each animal, a reference image with an interplane spacing of 2 μm was collected under light anesthesia (Isoflurane, ~1% by volume; Supplementary Fig. 2). Stacks were started just above the dura. Because the dura provides a reliably strong elevated fluorescent signal, we could automatically detect its appearance. The stack was divided into a 5-by-5 grid in the imaging plane, and dura depth was determined for each segment of the grid. The resulting points were fit to a plane using the singular value decomposition and this plane was used as the surface of the brain. For each point in the reference stack, depth was assigned based on the distance along a line through the point and perpendicular to the dural surface plane. This corrected for the fact that the objective’s image plane was typically tilted with respect to the dural surface by several degrees.

As described above, we used a single reference plane, typically from the first day of imaging, to align our imaging during subsequent sessions. We fit this reference image to the stack volume using a thin plate spline fitting algorithm. This allowed us to obtain a common (x, y, depth) coordinate for each pixel in the plane and, by extension, assign a depth to each recorded neuron.

The L1-L2 border was defined as the depth of the most superficially imaged excitatory neuron. The L3-L4 border was found by manually locating a noticeable shift in neuron morphology in conjunction with the emergence of clearly visible septa. The L2-L3 border was placed at the midpoint between the L1-L2 and L3-L4 borders. L5 neurons were excluded from analysis, and only appeared in a few mice. Because laminar boundaries are not discrete, analyses comparing layers were performed while excluding neurons within a 50 μm slice centered on the laminar boundary. This was done for the L2-L3 border and the L3-L4 border. Layers were typically thinner than observed anatomically^7^, likely due to compression from the cranial window.

Normalized depth was obtained by assigning a depth of 0, 1, and 2 to the L1-L2, L2-L3, and L3-L4 borders, respectively. Since L2 and L3 were of equal thickness, this was just a simple rescaling from the depth of the L1-L2 and L3-L4 borders to 0 and 2. Neurons in L4 were assigned a normalized depth using: 2 + (distance to L3-L4 border)/(distance from L2-L3 border to L3-L4 border).

### Resolution of overlapping signal sources

Source contamination due to pixels containing multiple neurons is a concern in two-photon calcium imaging^67^. This is especially problematic in the axial direction when a neuron appears across multiple planes. To identify such duplicates and to ensure that our data did not include multiple instances of the same neuron, we manually inspected all instances of candidate neuron pairs within a cylinder of radius 20 μm and half-height 40  μm that had a correlation above 0.2, keeping the neuron with the strongest signal and removing the others in instances where there were multiple candidates per real neuron. This resulted in the removal of 2835 ± 962 candidate neurons per mouse (mean ± S.D.; *n* = 7 mice).

### Encoding model and neural classification

Neurons were classified based on how well an encoding model could predict their activity on specific trial types (Supplementary Fig. 4). For each neuron, data across all imaged days was collated and fit simultaneously. The model predicts neural activity (*Δ*F/F), *r*_*model*_, from

$${r}_{{model}}=d \cdot \left[\left({j}_{{w}_{2}{w}_{1}}{a}_{{w}_{1}}+{j}_{{w}_{1}{w}_{2}}{a}_{{w}_{2}}\right)*\, g\right]+{\sigma }^{2}$$

Where ${a}_{{w}_{i}}$ is the predicted amplitude of response to a given whisker at a given time, *g* is the GCaMP kinetics kernel for that neuron, *d* is a session-specific scaling factor, *j* is a cross-whisker interaction term, and *σ*^2^ is a Gaussian noise term. For single-whisker touch trials for whisker *i*, ${a}_{{w}_{i}}$ is

$${a}_{{w}_{i}}={s}_{{pro}}\cdot {{lo}g}_{10}\left(-{\varDelta \kappa }_{{pro}}+{o}_{{pro}}\right){+s}_{{ret}}\cdot {{\log }}_{10}({\varDelta \kappa }_{{ret}}+{o}_{{ret}})$$

This model is based on previous work using a less constrained generalized linear model that revealed monotonically increasing response as a function of whisker curvature across touch neurons^30^. For a given whisker, the amplitude of the response to a protraction touch (*Δκ* < 0) at a given time,$\,{a}_{{w}_{i}},$ is given by applying a slope *s*_*pro*_ to its change in curvature, *Δκ*_*pro*_. To account for neurons that have a minimal force needed to elicit a response, the offset term *o*_*pro*_ was included. The retraction (*Δκ* > 0) response is calculated in an analogous manner.

The indicator kinetics kernel, *g*, consisted of a sum of exponentials having time constants *τ*_*rise*_ and *τ*_*decay*_. It was normalized so that its peak was 1. Both *τ*_*rise*_ and *τ*_*decay*_ were constrained based on the known physiological range^68^: *τ*_*rise*_, 100 ms to 500 ms; *τ*_*decay*_,1 s to 5 s. The noise term *σ*^2^ was determined for each neuron by measuring the variance of negative *Δ*F/F values. Our sliding-window F_0_ fitting procedure^36^, in which we compute F_0_ using a 3 min sliding window as the median for neurons that have low activity (non-skewed F distribution) and the 5th percentile for the most active neurons (highly skewed F distribution) ensures that *Δ*F/F is appropriately 0-centered.

Data were collected across multiple imaging sessions. To allow for some drift across sessions, we let the linear scaling factor *d* assume a single, unique value per session. Across all sessions, the maximal value of *d* was set to 1. For all other sessions, *d* reached a value between 0 and 1. This was designed to absorb variation in response across the multiple days of imaging.

The model was fit with 5-fold cross validation using block coordinate descent and a mean-square-error cost function minimizing the difference between model response, *r*_*model*_, and neural response, *r*_*neural*_. During cross-validation, data was partitioned by randomly drawing 5 disjoint equal sized sets of trials; individual trials were not broken up. The terms of ${a}_{{w}_{i}}$ were iteratively fit along with *g* and *d* using single-whisker touch trials and an equal number of non-touch trials. Because this resulted in two estimates of *g* and *d*, we employed the mean of these parameters for the final model fit; manual inspection revealed that the two individual whisker fits predicted similar values for these terms.

Following the single-whisker fits, a second fitting procedure was run to fit the interaction terms ${j}_{{w}_{1}{w}_{2}}$and ${j}_{{w}_{2}{w}_{1}}$ using the trials where both whiskers touched. Multi-whisker touch trials were classified based on the first two touches, and the scaling factor was applied to all touches by the second-touching whisker on that trial. Only trials where the interval between these first two touches was < 200 ms were included; this typically included the majority of multi-whisker trials (Supplementary Fig. 5a). For any trial where whisker 1 touched first, ${j}_{{w}_{1}{w}_{2}}$ was allowed to vary from 0 to 10, with values below 1 corresponding to suppression of the whisker 2 response and values above 1 corresponding to enhancement. An analogous procedure was used for trials where whisker 2 touched first.

The output of the full model, *r*_*model*_, was a predicted *Δ*F/F trace. In addition, a shuffled fit was performed in which the *Δ*F/F was shifted temporally, with wrap-around, by a random number of timesteps (minimum: 10 s, the approximate length of a trial) while leaving the *Δ*κ vectors untouched. A single shuffled fit was performed per neuron. To obtain a distribution of shuffled fits, neurons in a subvolume were grouped into 10 equal bins based on their calcium event rate, and neurons in a given bin used all shuffled fits in that bin. We classified neurons by using the Pearson correlation of the model-predicted and actual *Δ*F/F traces for specific trial types. Thus, a unidirectional single-whisker neuron would be one whose *Δ*F/F trace and model-predicted *Δ*F/F trace had a correlation for a single touch direction’s trials that met two criteria: correlation in excess of 0.1 and exceeding the 99th percentile of shuffled data correlation for event rate matched neurons. Neurons were classified as unidirectional single-whisker neurons if they only met criteria when the Pearson correlation was calculated for one single-whisker touch type (W1P, W1R, W2P, or W2R). Neurons meeting criteria for both touch types for a single whisker were classified as bidirectional single-whisker. Neurons were classified as multi-whisker if they met our criteria for at least one direction for each whisker.

### Matching L2/3 to L4 imaging noise levels

We adjusted L2/3 ∆F/F traces to approximate the L4 signal-to-noise ratio (SNR; Supplementary Fig. 6). First, we computed the mean of the top 1% of ∆F/F values for each cell in L2/3 as well as the mean of these values for L4 for each animal. This ratio, *α*_*L4-matching*_, was used to linearly scale L2/3 ∆F/F. Next, produced Gaussian noise matching that observed in L4 (measured by fitting negative ∆F/F values in L4 to a Gaussian) and added the resulting vector, σ _L4-matching_, to L2/3 ∆F/F responses. adding a noise term, σ_L4-matching_ (Methods).

### Response probability analysis

Neurons were classified as responsive or non-responsive for every touch trial by comparing the post-touch *Δ*F/F to the baseline *Δ*F/F. Baseline *Δ*F/F was calculated as the mean *Δ*F/F for the 6 frames (0.85 s) preceding the first touch. The post-touch *Δ*F/F was calculated as the mean *Δ*F/F for the period between the first touch and two frames after the final touch. For each neuron, we obtained a noise estimate by fitting all negative *Δ*F/F values to a half-normal distribution, yielding a noise term, σ. Neurons were considered responsive on a given trial if the *Δ*F/F_post-touch_ > *Δ*F/F_baseline_ + σ and if *Δ*F/F_post-touch_ exceeded the 99^th^ percentile of shuffled *Δ*F/F_post-touch_ values for that trial. Shuffled *Δ*F/F_post-touch_ was calculated by temporally shifting the *Δ*F/F vector, with wrap-around, by a random number of timesteps (at least 10 s, the approximate length of a trial). This was done 100 times per neuron, yielding a distribution of shuffled *Δ*F/F_post-touch_ values for each neuron and touch trial. Neurons that were responsive on at least 10% of trials for a single touch type were considered part of the responsive pool.

### Decoding analysis

Single neuron decoding was performed by computing the post-touch *Δ*F/F across two sets of trials. We used two trial partitioning schemes. For force decoding, single-whisker trials of a single type (W1P, W1R, W2P, W2R) were divided into equal-sized thirds based on mean trial *Δ*κ. The ability of a neuron to distinguish between the top third (‘strong’) and bottom third (‘weak’) of trials based on *Δ*κ was evaluated. For whisker identity decoding, all single whisker touch trials for whisker 1 were compared to single whisker trials for whisker 2.

For each trial belonging to the pair of trial types under examination, we computed the mean *Δ*F/F between the first touch and first lick; if the first lick occurred > 2 s after the first touch, only 2 s after the first touch were used. Receiver operating characteristic (ROC) analysis was performed by sliding a criterion threshold through the range of *Δ*F/F values across the two trial types. We report the area under the curve (AUC) resulting from this analysis^69^. Decoding was performed only if at least ten trials of each type were present.

### Correlation analysis

Pearson correlations were calculated across neuron pairs for each layer using the subvolume that had the most neurons belonging to that layer for that particular animal. We measured correlations either over all time points, or restricted to time points outside of touch. This was defined as excluding any timepoints 1 s prior to and 10 s after a touch. When computing correlations, single-whisker neurons were grouped by subtype. That is, for unidirectional single-whisker contacts, we independently computed a mean correlation for each animal (and, for distance analysis, for a given distance) among W1P, W1R, W2P, and W2R neurons. The mean of these was then used as the correlation for unidirectional single-whisker neurons. A similar process was used for bidirectional single-whisker neurons, with grouping by whisker. For multi-whisker neurons, we did not break up by subtype, as the number of neurons of a given subtype was too low.

### Ensemble detection

As with correlation and decoding analysis, the subvolume with the most neurons for a given layer was used in analyzing ensembles for that layer, allowing for analysis of concurrently recorded neurons. Hierarchical clustering was performed on the correlation matrix computed for all time points to identify small (3–5 neuron) groups of neurons with high mutual pairwise correlations. Each cluster was used as a seed for the greedy algorithm, which at each step added the neuron that had the highest mean correlation with the existing ensemble members. The process continued until no neuron could be added without reducing the mean within-ensemble correlation below a threshold defined as twice the 99.5th percentile of all correlation values. Because this produced many redundant ensembles, ensembles with at least 75% overlap were merged provided this would not violate the minimum correlation criteria. Ensembles with less than 3 members were discarded; such ensembles were rare, and never showed touch-related activity. The percent of touch neurons in an ensemble was most often either ~0% or ~100% (Fig. 7b); therefore, touch ensembles were defined as those ensembles for which at least 50% of neurons were classified as touch neurons by our encoding model.

### Statistics and reproducibility

For comparisons across two matched groups, the two-sided, paired t-test was used. Typically, pairing was within-animal. Multiple (3 or more) groups were compared using a one-way ANOVA. In cases where this yielded significance (*p* < 0.05), post-hoc Tukey’s Honestly Significant Difference (HSD) test is reported for comparisons between pairs of groups.

### Reporting summary

Further information on research design is available in the Nature Research Reporting Summary linked to this article.

## Supplementary information

Supplementary Information

Reporting Summary

## Data Availability

The data generated in this study is available at: http://peronlab.org/data/2022_voelcker_et_al_two_whisker.zip. Source data are provided with this paper.

## Source data

Source Data

## Code availability

Code used to analyze the data and generate the figures is available at the following Github repository: https://github.com/peronlab/two_whisker_voelcker_2022.
