# Supplementary material for: Transformation of primary sensory cortical representations from layer 4 to layer 2
Source: Nat Commun. 2022 Sep 19;13:5484. doi: 10.1038/s41467-022-33249-1 (PMC9485231; doi:10.1038/s41467-022-33249-1)
Supplement: Supplementary file 1 — Supplementary Information [file 41467_2022_33249_MOESM1_ESM.pdf]

Animal ID	Sex	Age at Training Start	Spared Whiskers	Number of Subvolumes	L2 Cell Count	L3 Cell Count	L4 Cell Count
**274688**	M	67d	C1, C2	7	5,431	6,833	5,351
**279029**	M	73d	C2, C3	5	3,140	6,370	4,608
**279608**	F	59d	C2, C3	5	1,927	3,776	5,689
**280201**	M	81d	C2, C3	6	4,152	4,512	6,792
**283544**	M	93d	C2, C3	5	3,844	5,098	5,672
**284891**	F	60d	C2, C3	5	3,818	6,688	4,703
**284893**	M	63d	C1, C2	5	4,263	4,205	4,788
**014261**	M	93d	C2,C3	2	1,631	1,978	1,238
**014347**	F	68d	C2,C3	2	2,412	2,475	718
**014348**	F	68d	C2,C3	2	895	1,120	1,032
**015144**	M	86d	C2,C3	2	1,489	1,575	1,151
**015741**	M	75d	C2,C3	2	2,188	1,935	536

Supplementary Table 1. List of animals. All mice were transgenic Ai162 X Slc17a7-Cre, expressing GCaMP6s in excitatory neurons. Age is given in days. Unlike for most analyses, where neurons in a 50 µm slice centered on the layer boundary were omitted, provided cell counts include all neurons imaged. The first seven animals comprise the core dataset used in the paper. The final five animals were used to control for effects of trimming and training (**Supplementary Figure 7** only).

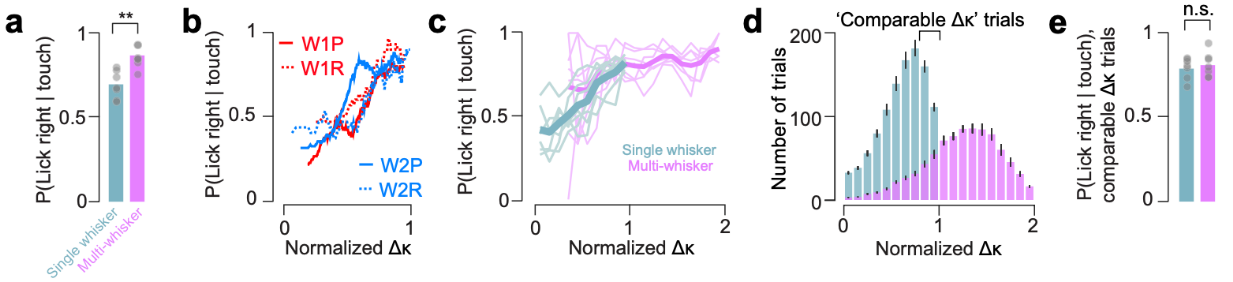

Supplementary Figure 1. Probability of reporting touch on single versus multi-whisker touch trials. a. Mean probability of licking right for all single whisker and multi-whisker touch trials across all mice (n=7). **, $p < 0.01$, two-sided paired t-test comparing performance across mice. **b.** Example mouse probability of licking right on single whisker touch trials as a function of touch force for all four single whisker touch types (normalized Δκ values are normalized to the 95th percentile of Δκ values; Methods). **c.** Probability of licking right as function of touch force for both single whisker and multi-whisker touch trials across all mice. Thick lines, means; thin lines, individual animals. Normalized Δκ values were summed for both whiskers. **d.** Number of trials of each type across normalized Δκ bins across mice (n=7). Bars show mean ± S.E.M. (n=7 mice). **e.** Mean probability of licking right for single whisker and multi-whisker touch trials matched for normalized Δκ (using trials with values between 0.8 and 1; n=7 mice); n.s.: not significant.

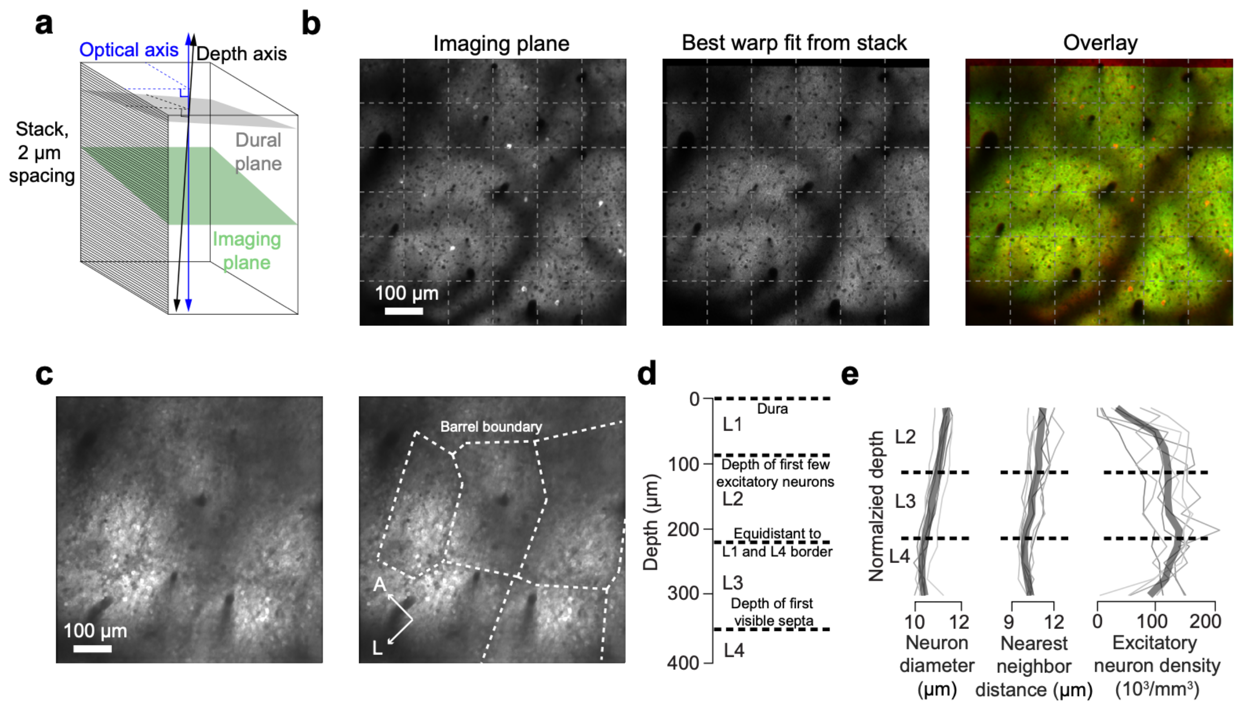

Supplementary Figure 2. Assignment of cortical depth. a. A reference stack with 2 µm spacing is used to assign cortical depth. Depth is calculated with respect to the axis perpendicular to the dural plane (grey; Methods). The imaging plane (green) is then aligned to the reference stack, allowing for an assignment of depth to each imaging plane pixel. **b.** Example thin plate spline warp field fit. Left, the imaging plane. Middle, best fit obtained from the stack. Due to anesthesia, activity is reduced in the stack plane. Right, overlay of both. **c.** Example septa in L4; appearance of septa was used as criterion for determining L3-L4 border. Left, raw image. Right, barrel boundaries inferred from septa. **d.** Method for the assignment of each laminar border. **e.** Morphological parameters as a function of normalized depth. Light lines, individual animals; dark thick line, cross-animal mean. Left, mean diameter of cells at a given depth. Middle, distance for each neuron to its nearest neighbor. Right, volumetric density of excitatory neurons.

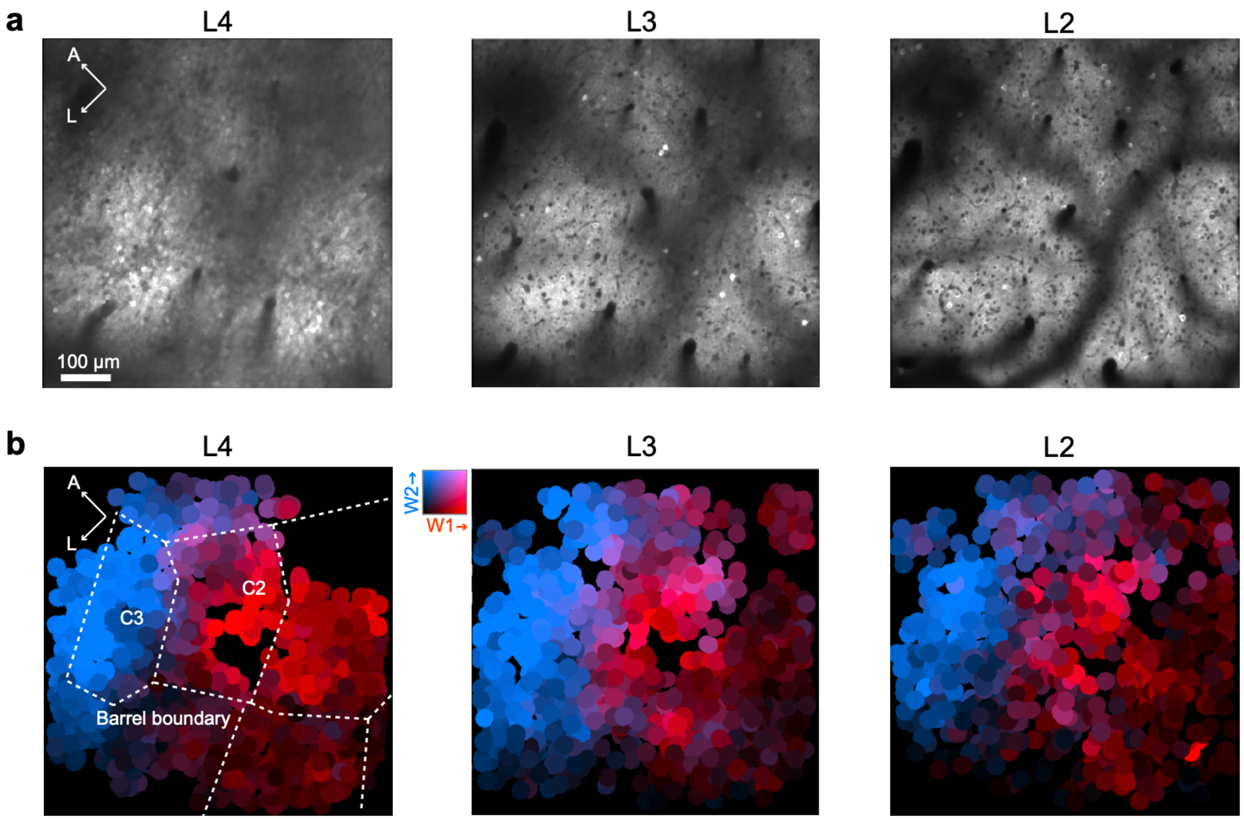

Supplementary Figure 3. Barrel identification. a. Example planes from L2, L3 and L4. **b.** Neuropil signal centered at each soma. The neuropil signal was computed for all pixels within 3-13 µm away from the neuron border, excluding any pixels belonging to a soma or those with neighbor pairwise correlation exceeding 0.2. Color code indicates sensitivity to whisker 1 (red) or whisker 2 (blue); in this case, the C2 and C3 whiskers. Barrel boundaries from L4 in **a** are traced in the whisker map.

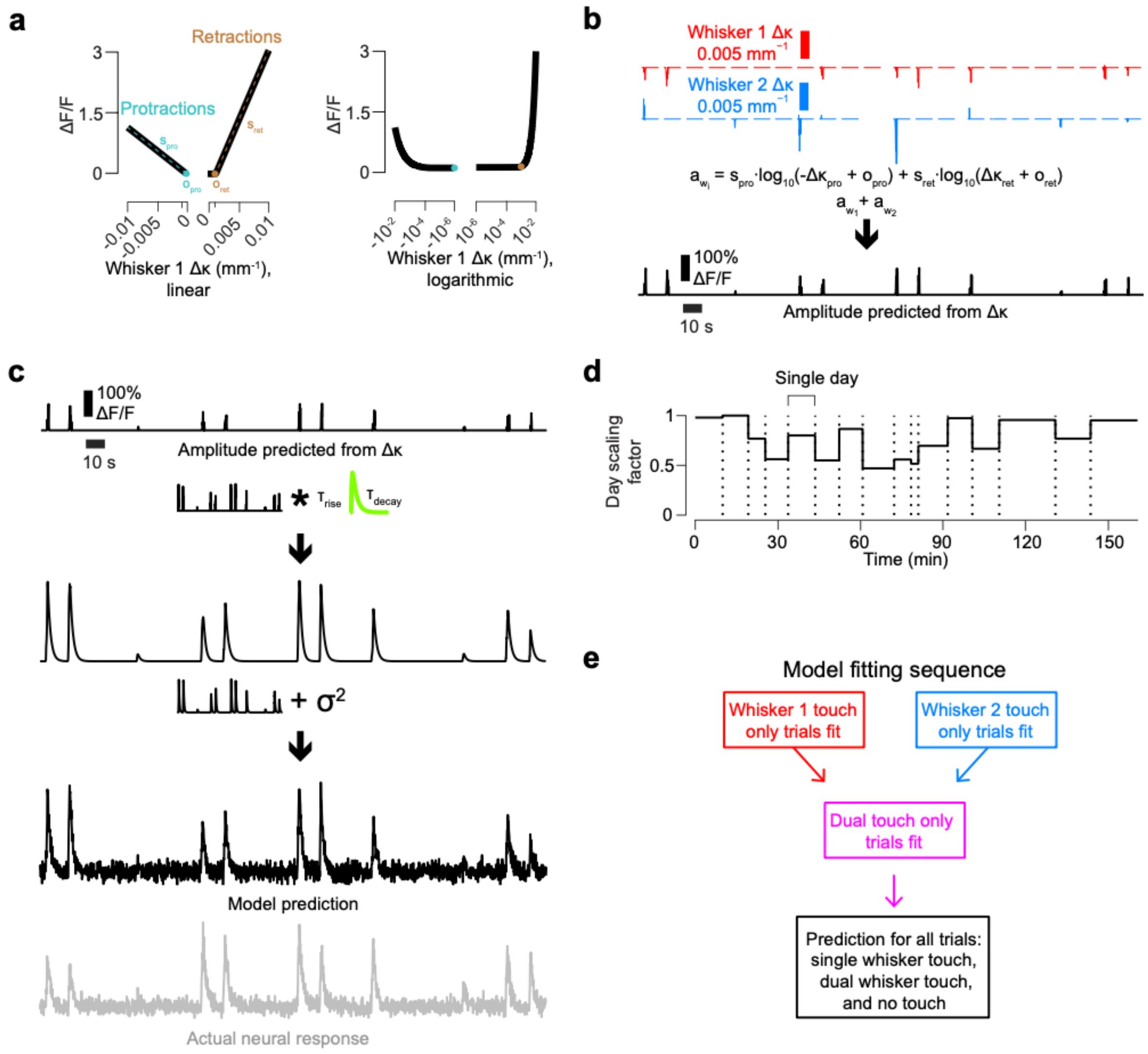

Supplementary Figure 4. Encoding model. a. Example Δκ kernel, which maps instantaneous Δκ to a ΔF/F amplitude. If the cell has a Δκ threshold for that particular direction of touch, o_{ret} or o_{pro} will be non-zero. Right, logarithmic plot. **b.** The individual kernels are applied to the relevant Δκ trace to produce a_{w_i} for each whisker; these are summed to produce the overall ΔF/F amplitude prediction. **c.** The amplitude prediction from **b** is convolved with a GCaMP6s kinetics kernel, which is a sum of exponentials (Methods). Next, noise is added (σ^2; Methods), resulting in the full prediction. **d.** Example day scaling factor applied over the imaging days. **e.** Fitting procedure for exclusive touch model.

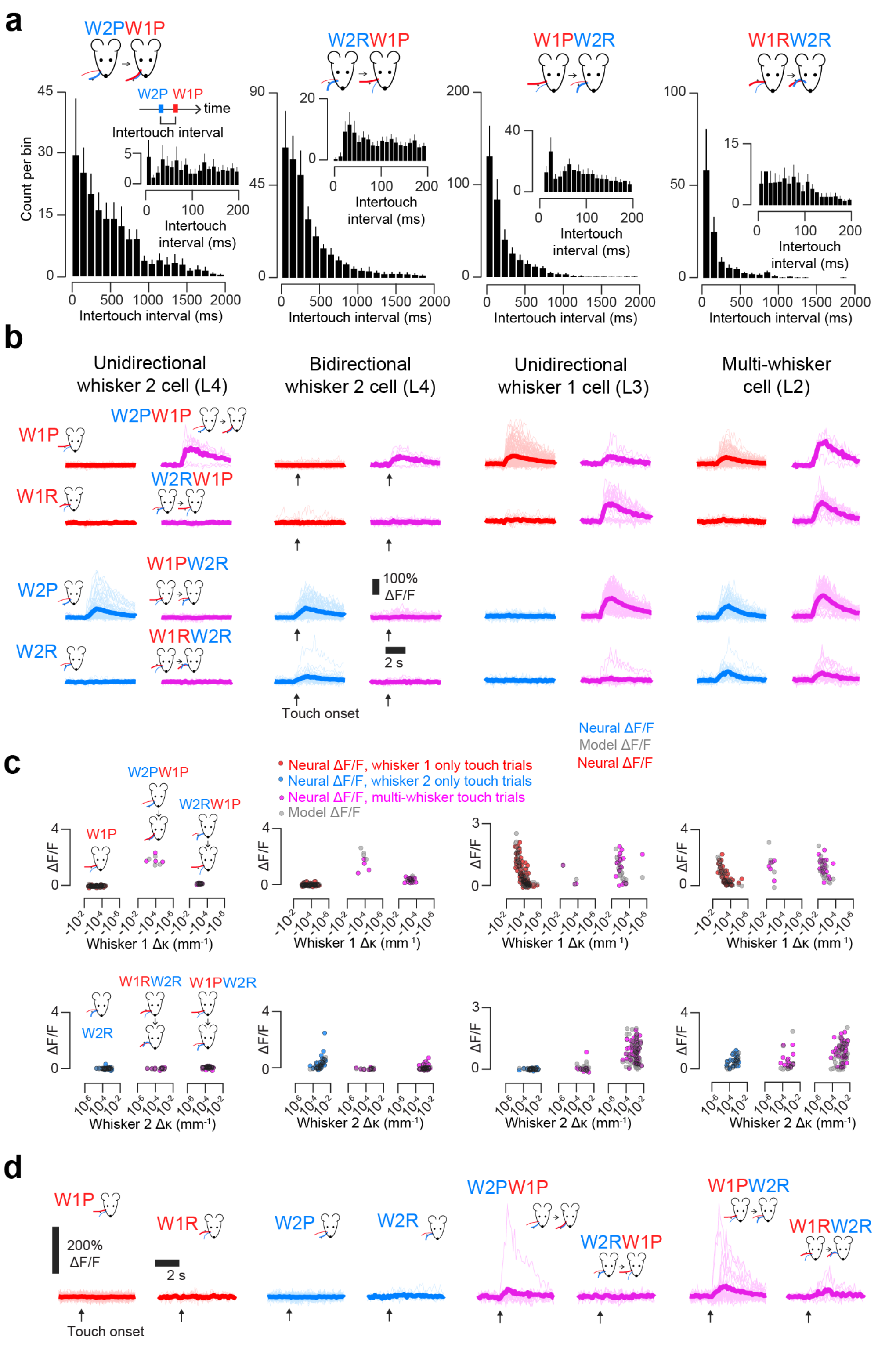

Supplementary Figure 5. Responses on multi-whisker touch trials. a. Inter-touch interval distributions for the four multi-whisker touch types (W2PW1P, W2RW1P, W1PW2R, W1RW2R). The interval is between the first two touches on any multi-

whisker trial; 100 ms bins were used, with inset showing 10 ms bins for first 200 ms. Bars show mean ± S.E.M. (n=7 mice). Plot includes all touches for a given mouse. **b.** Example ΔF/F responses to four single-whisker and four multi-whisker touch types for four neurons. Light color, individual touch-aligned responses; dark color, mean across touches. Traces are colored according to touch type, indicated above the traces. **c.** Comparison of actual vs. model tuning curves. The mean ΔF/F as a function of Δκ for each trial is shown with colored circles. Red circles, trials where only whisker 1 touched; blue, only whisker 2 touched; purple, both whiskers touched. Gray circles, model's predicted ΔF/F for the same trials. For multi-whisker touch trials, Δκ is given for the second whisker that touched. By convention, Δκ is negative for protraction touches. **d.** One of two neurons across the entire dataset that showed no response to single whisker touches but substantial responses to multi-whisker touches.

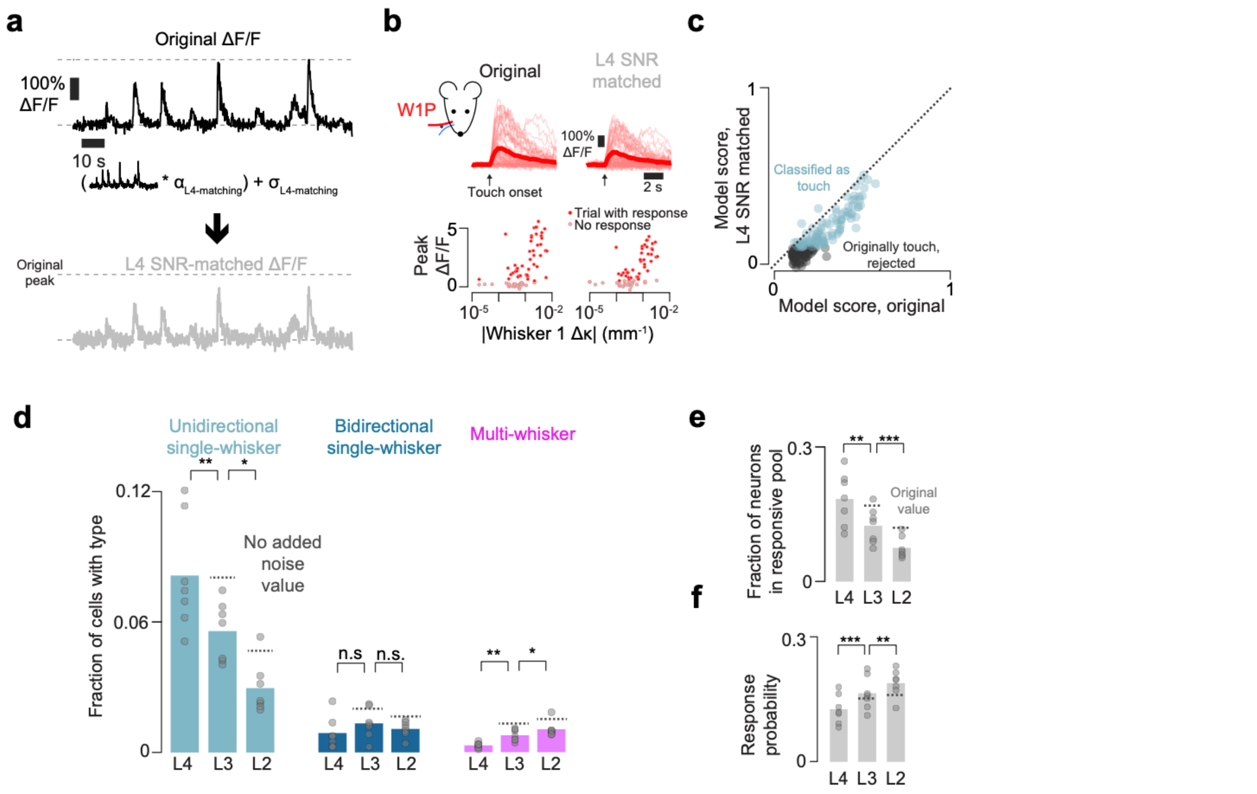

Supplementary Figure 6. Touch cell distribution after L4 SNR matching in L2/3. a. Signal-to-noise ratio (SNR) in superficial layers was matched to that in L4 by adjusting the response peak by a multiplicative term $\alpha_{L4\text{-}matching}$ and adding a noise term, $\sigma_{L4\text{-}matching}$ (Methods). **b.** Example L2 neuron responses to whisker 1 protractions before and after L4 SNR matching. **c.** Encoding model score for single whisker unidirectional touch neurons before and after SNR matching. Blue, cells that were classified as touch following adjustment; black, cells that were no longer classified as touch cells. **d.** Distribution of touch cell types by layer across dataset following SNR matching (see **Fig. 3b**). Grey dashed lines, original values for L3 and L2. Bars indicate mean across mice (n=7). P-values indicated for paired t-test, *: $p < 0.05$; **: $p < 0.01$; ***: $p < 0.001$. **e.** Mean response pool size following SNR matching across mice. **f.** Mean response probability for response pool members for each layer across mice.

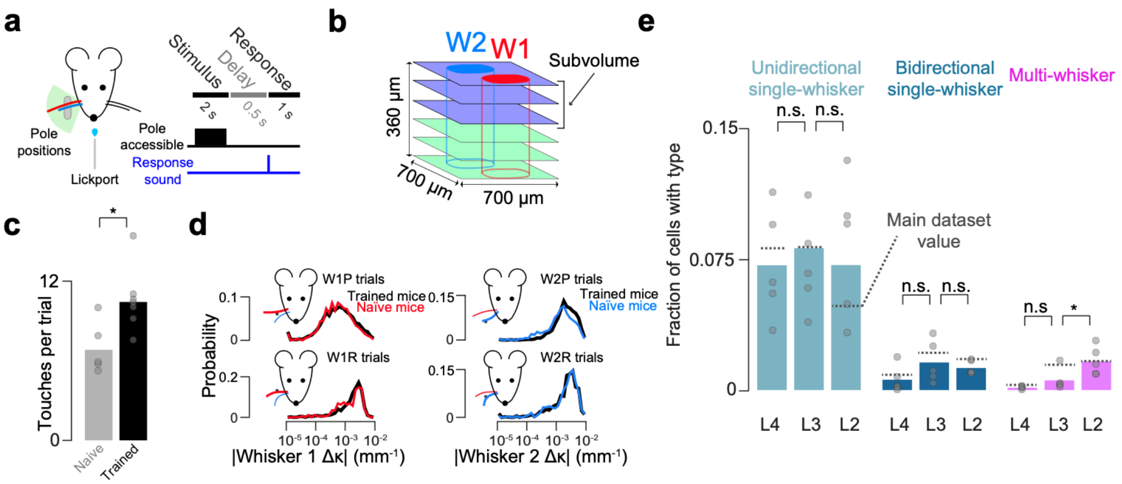

Supplementary Figure 7. Touch cell distribution in recently-trimmed, task-naïve mice. a. Mice were trimmed then immediately started on a single lickport variant of the task where all trials had an accessible pole and were rewarded (Methoxds). **b.** Two subvolumes each spanning 180 µm were imaged over 2-3 days. **c.** Mean number of touches on trials with at least one touch for naïve (n=5) and trained (n=7) mice. P-values indicated for paired t-test, *: $p < 0.05$; **: $p < 0.01$; ***: $p < 0.001$; n.s.: not significant. **d.** Distribution of contact forces in naïve mice. **e.** Distribution of touch cell types by layer in naïve mice. Dashed line, value from main dataset. Bar indicates mean across mice (n=7).

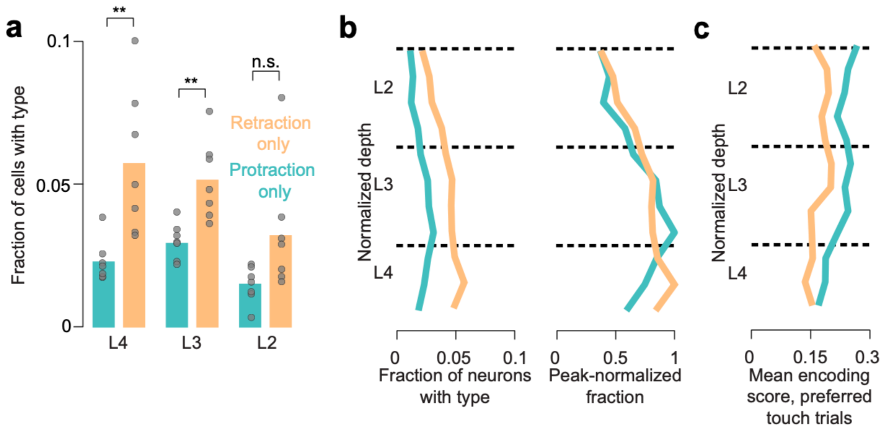

Supplementary Figure 8. Laminar distribution of unidirectional single-whisker cells by directional preference. a. Frequency of protraction and retraction preferring unidirectional single-whisker cells for L4, L3, and L2. Bars show mean (n=7 mice). P-values indicated for paired t-test, *: $p < 0.05$; **: $p < 0.01$; ***: $p < 0.001$; n.s.: not significant. **b.** Distribution of directional cell types as a function of normalized laminar depth (Methods). Left, fraction of cells at a given laminar depth. Right, Normalized fraction. **c.** Encoding score for given directional cell type as a function of normalized depth. Encoding score was calculated only across trials of the preferred type (i.e., trials with touches to which the neuron was significantly responsive; Methods).
